# Physical activity, sedentary behaviour and screen time among youths with Down syndrome during the COVID‐19 pandemic

**DOI:** 10.1111/jir.12933

**Published:** 2022-04-21

**Authors:** S. Amatori, D. Sisti, F. Perroni, G. Brandi, M. B. L. Rocchi, E. Gobbi

**Affiliations:** ^1^ Department of Biomolecular Sciences University of Urbino Carlo Bo Urbino Italy

**Keywords:** Down syndrome, intellectual disability, physical activity, parenting, sedentariness, young adults

## Abstract

**Background:**

The COVID‐19‐related restrictions hampered habitual physical activity (PA), particularly affecting the more vulnerable, such as people with Down syndrome (DS). The study aimed to investigate changes in PA, sedentary behaviour (SB) and screen time (ST) of youths with DS, before, during and after the restrictions, also in relation to parental PA levels.

**Methods:**

A cross‐sectional design with a retrospective assessment of variables for the before and during restrictions periods was adopted. Parents of youths with DS completed an online questionnaire. Sociodemographic aspects, weekly PA levels and youths' daily SB and ST were investigated, referring to three time‐points: before the pandemic, during the restrictions and the restrictions‐easing phase.

**Results:**

A total of 57 parents voluntarily participated in the study, proxy‐reporting on their child (male = 41, female = 16, age = 21.4 ± 7.7 years). A repeated measures multivariate analysis of variance showed negative effects of restrictions (*P* < 0.05) on PA levels, SB and ST, independently from sociodemographic characteristics. In the restrictions‐easing phase, PA levels did not return to before the pandemic values (*P* < 0.05). A positive correlation between parents and their child's PA was detected before the pandemic (*r* = 0.38; *P* < 0.01), no longer reported in the restrictions‐easing phase.

**Conclusions:**

The findings showed the negative impact of restrictions on youths with DS lifestyle. Moreover, the importance of addressing the needs of the disabled community including the whole family is highlighted.

## Introduction

COVID‐19 affected the whole world causing a crisis in the health, economic, education, political systems and social life of all countries (Settersten *et al*. 2020). People with intellectual disability (ID) have been reported to have a higher risk of mortality associated with COVID‐19 infection than those without ID. In particular, people with Down syndrome (DS) might experience more severe symptoms at hospitalisation (e.g. confusion) and higher rates of lung complications (Huls *et al*. 2021). Other adverse outcomes include a possible exacerbation of psychiatric conditions and worsening of functional and cognitive impairment (Doody & Keenan 2021). In the second instance, concerns are also related to the burden generated by restrictions and strict social isolation measures implemented by several countries to contain the pandemic spread. Because of these public health measures, it was more challenging than usual to continue habitual physical activity (PA) (Stockwell *et al*. 2021). Particularly, PA levels decreased with a consequent increase in sedentary behaviour (SB), screen time (ST) and poor dietary habits both in the general population (Amatori *et al*. 2020; Nagata *et al*. 2020) and in people with a disability (Courtenay 2020; Tummers *et al*. 2020). PA (including sport and exercise) offers important health benefits on populations with a high risk of comorbidities, such as people with ID (Carraro & Gobbi 2014; Kapsal *et al*. 2019) and more specifically with DS (Andriolo *et al*. 2005; Ptomey *et al*. 2018; Paul *et al*. 2019). In normal conditions, individuals with DS show lower fitness and PA rates (Baynard *et al*. 2008), do not meet PA guidelines across age groups (Fox *et al*. 2019) and show a high prevalence of sedentariness (Esposito *et al*. 2012; Oviedo *et al*. 2017) compared with the general population. Thus, because of PA central health‐protective and promoting effects, it is important to evaluate the adjunctive impact of the pandemic restrictions on PA level among people with DS.

Gender, ID level and residential type/location have been reported as significant correlates of PA‐related behaviours. Namely, being a female, having a higher ID level, and living in rural areas with reduced services and lack of transportation are generally associated with worsened levels of PA and SB (Temple 2010; Oviedo *et al*. 2019; Westrop *et al*. 2019; Hsu *et al*. 2021). Within the social domain, parents/caregivers have been shown to exert considerable influence over their child's PA (Schor and American Academy of Pediatrics Task Force on the Family 2003). In particular, the parent's PA directly influences the child's PA (parent modelling) both in the general (Loprinzi *et al*. 2013) and ID population (Siebert *et al*. 2017). Furthermore, people with disabilities are often dependent on their parents/caregivers as they may be unable to take advantage of formal and informal PA opportunities, such as going to the gym or playing in a neighbourhood park (Bodde & Seo 2009; Martin & Choi 2009). During the COVID‐19 pandemic, parents of children with disabilities, concerned by their child's vulnerability, might have become more overprotective, exacerbating social isolation and physical inactivity (Embregts *et al*. 2021). Hence, parents/caregivers were considered a key element in exploring PA and SBs of youth with DS in the transitions through the COVID‐19‐related restrictions.

Within this background, understanding PA and SB trends in youths with DS during the pandemic‐related restrictions is important to help inform policymakers about possible counteracting strategies. Nevertheless, few data are available about lifestyle patterns variability and the impact of restrictions within this population (Villani *et al*. 2020; Theis *et al*. 2021). To the best of our knowledge, there is no evidence yet on this topic. Therefore, the primary aim of the present study was to describe the changes in PA habits, sedentary and screen time due to COVID‐19‐related restrictions in a sample of youths with DS considering personal characteristics as fixed factors. As a secondary aim, the associations between pre‐pandemic and restriction‐easing PA levels were assessed within groups to explore to what extent youths and parents returned to their habitual PA and between groups to account for parental modelling.

## Methods

### Procedures

Parents/caregivers of young people with DS affiliated to three different family associations for people with disability were invited to complete a 15‐min‐long questionnaire administered via online survey platforms (i.e. Google Forms) and voluntarily accessed by participants using a designated link. The online survey was accessible for 2 weeks from 21 May 2021, corresponding to the restrictions‐easing phase in Italy. The study was developed in accordance with the principles embodied in the Declaration of Helsinki for the protection of human rights, and it was exempted from the institutional review board approval. All the participants gave their electronic informed consent before being directed to the survey. The research procedures were clearly explained, and participants could interrupt or quit the survey at any point before the submission without explaining the reasons for doing so, avoiding the storage of their data. Neither participant's name nor contact information were asked. By clicking the ‘submit’ button at the end of the questionnaire, the participant's answer was stored and then used for the resulting data.

### Participants

Fifty‐seven parents (49 mothers and 8 fathers from different families, 56.0 ± 7.9 years old) completed the online questionnaire. With regard to their children, 41 were males and 16 females (age: 21.4 ± 7.7 years; height: 156.8 ± 11.2 cm; weight: 59.9 ± 15.9 kg; body mass index: 24.1 ± 4.7 kg/m^2^); 16 reported mild, 26 moderate and 15 severe ID levels. Eighteen of them lived in the city centre, 24 in the suburbs and 15 in rural areas; only one lived independently and the rest with their families (this factor was not considered for the analysis).

### Measures

The questionnaire comprised two main sections investigating *sociodemographic information* and *dependent variables of interest*. The latter comprised three subsections where participants were requested to answer the questions respectively referred to before the pandemic, during the restrictions and the period of restrictions‐easing. The following stem introduced the questionnaire subsections: ‘This part of the questionnaire focuses on your child's behaviours during the period …’ differentiating the three time‐points. This organisation led the parents to not directly compare their answers on the three time‐points, limiting a possible comparison bias.

#### Sociodemographic information

Sociodemographic data of parents/caregivers included gender and age. Moreover, they were asked about their habitual PA (expressed in hours/minutes per week) before the pandemic and during restrictions‐easing. PA was explained as ‘at least moderate‐intensity PA, including sport, exercise and leisure time PA’ (WHO 2020). Finally, they were asked to proxy‐report gender, age, height and weight (that served to calculate BMI), ID level (mild, moderate and severe), residential type (family, independent and community‐based) and location (city centre, suburbs and rural area), in order to capture possible sources of influence on PA and SB of their children. Moreover, to better describe possible PA changes, parents were asked about their child's sports practice (before the pandemic and during restrictions‐easing), also questioning motives that possibly impeded the return to the pre‐pandemic habits.

#### Dependent variables of interest

Dependent variables of interest comprised youths' habitual PA in a week, and habitual SB in a day (Owen *et al*. 2011), grouping two specific domains: time spent sitting/lying in domestic environments, in the workplace or transportations (e.g. while doing course work, visiting friends and reading), and screen time (TV viewing and other screen‐focused behaviours).

##### Proxy‐reported physical activity

To investigate habitual PA, the adaptation of the WHO‐HBSC item on PA weekly duration was used (Holmen *et al*. 2002). PA was explained as activities that take moderate or vigorous physical effort and make the person breathe somewhat harder than normal (Lee *et al*. 2011). Parents were asked to report hours and minutes of their child's PA by answering the following item ‘On a typical week, how many hours/minutes did your child usually exercise in his/her free time, so much that he/she gets out of breath or sweat?’. Proxy‐reported PA was analysed both as raw data, and by considering the WHO guidelines for people with disability, in order to identify participants who did respect or did not the guidelines. WHO guidelines recommend thresholds of 60 min/day for under 18 and 150 min/week for adults (when not contraindicated) (Bull *et al*. 2020).

##### Proxy‐reported sedentary behaviour and screen time

To investigate SB, the last question of the International Physical Activity Questionnaire Short Form (Lee *et al*. 2011) was adapted and used. Firstly, SB was defined as the time spent sitting/lying at home, at work and during leisure time while reading, eating and doing homework, as a passenger in a motor vehicle. Parents/caregivers were then questioned referring to the three conditions: ‘On a typical day, how much time in hours/minutes does your child spend in sedentary behaviours?’. In addition, parents recorded how much time their child engaged in specific SBs, as follows: ‘On a typical day, how many hours/minutes did your child spend awake in a room with the television on, videos or a DVD on, playing the computer, video game consoles, or handheld devices?’. Proxy‐reported screen time (ST) was used as both raw data and, by considering the international recommendation of a maximum of 2 h/day (American Academy of Pediatrics: Committee on Public Education 2001), to group participants who respected or exceeded the recommendation.

### Statistical analysis

Descriptive statistics (means and standard deviations; percentage distributions) were conducted to describe the sample characteristics. To assess the mean differences of youths' PA, SB and ST between before the pandemic, during restrictions and in the restrictions‐easing phase, a mixed between‐within repeated measures multivariate analysis of variance was performed, with three times (pre‐pandemic, during restrictions and restrictions‐easing), two genders (male and female), three ID levels (mild, moderate and severe) and three living area categories (city centre, suburban and rural area) main effects design. Bonferroni correction test was used for post‐hoc pairwise comparisons. Simple contrasts were also calculated to check differences between times (during vs. pre‐pandemic, restriction‐easing vs. pre‐pandemic). Effect sizes were computed using partial eta square (*η*_p_^2^). Pearson's correlations were calculated between youths and their parents' PA levels, both in pre‐pandemic and restrictions‐easing, and within‐groups at the two time‐points. The analyses were performed using SPSS Statistics 26 (IBM, Armonk, NY, USA). The level of significance was set at *P* < 0.05.

## Results

### Qualitative description of physical activity, sports practice and screen time

Parents reported that almost all youths (95%) were involved in one or more sports activities before the pandemic, with only three not participating in any sport. Swimming (51%), soccer (21%), basketball (18%) and track and field (11%) were the most played sport. Half of the youths did not restart practising their sport in the restrictions‐easing phase, 30% of them resumed their sports with a lower frequency and only 20% returned to their sport with the same frequency as before the pandemic. The most common reasons they could not restart practising sports activities were related to the facilities that were still closed, specific courses within the facilities that did not begin again or parents' fear of possible contagion. Applying the thresholds for recommended PA (Bull *et al*. 2020), 35.1% of the participants respected the guidelines in the pre‐pandemic, a proportion that drastically decreased to 12.3% during the restrictions period, and partially moved up to 21.1% in the restrictions‐easing phase. According to the recommendation for ST (American Academy of Pediatrics: Committee on Public Education 2001), the prevalence of participants meeting the guidelines was 54.4%, 29.9% and 42.1% in the three time‐points, respectively. The prevalence of those meeting weekly PA, daily ST and both are reported in Table 1.

**Table 1:** Prevalence of youths with DS meeting weekly physical activity (PA) and daily screen time (ST) recommendations in pre‐pandemic, during restrictions and in restriction‐easing phase, expressed as *n* (% of the total sample)

	Pre‐pandemic		During restrictions		Restrictions‐easing	
	Meet PA	Do not meet PA	Meet PA	Do not meet PA	Meet PA	Do not meet PA
Meet ST	12 (21.1%)	19 (33.3%)	3 (5.3%)	14 (24.6%)	6 (10.5%)	18 (31.6%)
Do not meet ST	8 (14.0%)	18 (31.6%)	4 (7.0%)	36 (63.2%)	6 (10.5%)	27 (47.4%)

Thresholds for PA in accordance with the WHO guidelines (Bull *et al*. 2020); thresholds for ST in accordance with the American Academy of Pediatrics: Committee on Public Education (2001).

On the other hand, among the parents, 58% (33 participants) reported to be used to regularly practising PA or sport before the COVID‐19 pandemic (fitness activities, jogging and trekking were the most practised); notably, only 20 people reported to have restarted their usual practice in the restrictions‐easing phase, with almost two‐thirds of the parents reporting to be inactive in this phase due to a different organisation of their domestic and working routines.

### Restrictions' effects on physical activity, sedentary behaviour and screen time

Multivariate analysis of variance for repeated measures showed a significant effect of time on PA, SB and ST (Table 2), all the three with large effect sizes. All measures were significantly different during the restrictions period with respect to pre‐pandemic, with a halving of weekly PA and a substantial increase in daily SB and ST; notably, in the restriction‐easing condition, only PA remained significantly different from the pre‐pandemic condition (e.g. youths did not go back to their previous levels of PA), while both SB and ST returned to not significantly different levels than pre‐pandemic. No significant interaction effects were found between time and gender, ID level or living area, for none of the considered dependent variables (*P* > 0.05).

**Table 2:** Descriptives (expressed as mean ± standard deviation) and repeated measures multivariate analysis of variance results for participants with DS' physical activity, sedentary behaviour and screen time in pre‐pandemic, during restrictions and restrictions‐easing phase

	Pre‐pandemic	During restrictions	Restrictions‐easing	*F*_2,102_	*P*	*η*_p_^2^
PA (min/week)	226.8 ± 127.4	105.2 ± 132.0^a^	158.4 ± 134.1^b^	17.12	<0.001	0.25
SB (min/day)	196.8 ± 117.3	261.1 ± 142.3^a^	210.5 ± 123.2	12.06	<0.001	0.19
ST (min/day)	186.8 ± 141.7	251.6 ± 144.6^a^	203.2 ± 131.3	13.48	<0.001	0.21

PA, physical activity; SB, sedentary behaviour; ST, screen time.

^a^ Simple contrasts results: during vs. pre‐pandemic (*P* < 0.001).

^b^ Simple contrasts results: restrictions‐easing vs pre‐pandemic (*P* < 0.001).

### Correlation analyses

**Figure 1:**
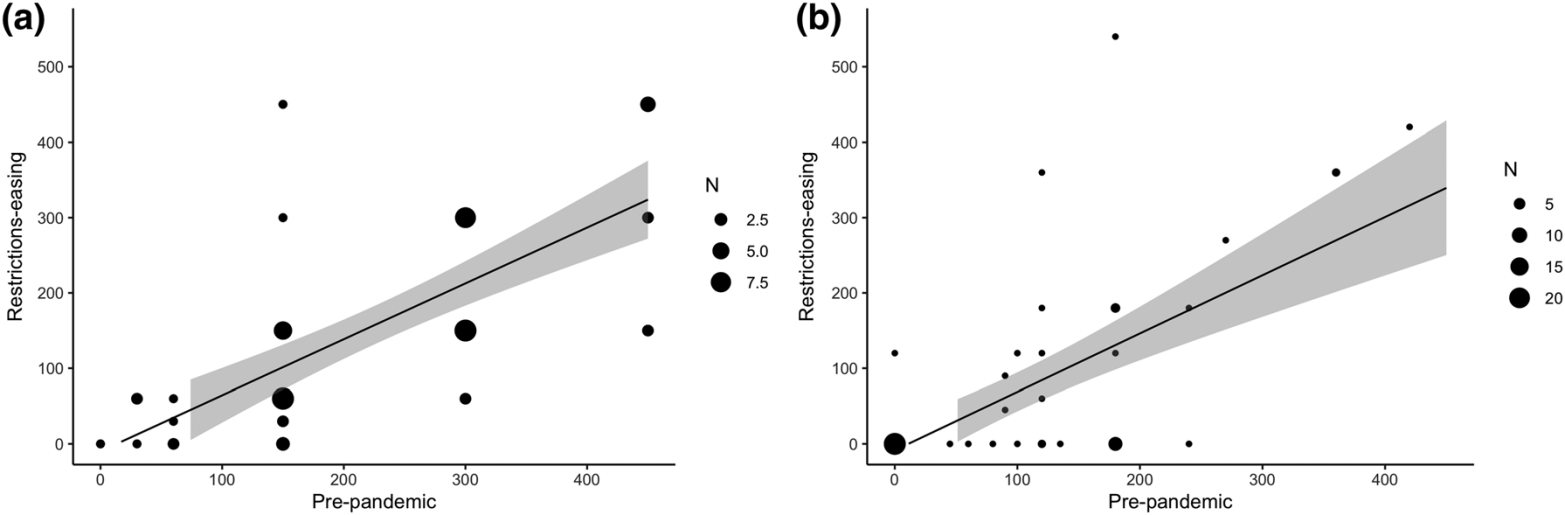
Correlations between PA levels (min/week) in the pre‐pandemic (*x*‐axis) and restrictions‐easing (*y*‐axis) conditions, for youths (a) and parents (b). The size of the circles gets bigger for overlapped points.

Within‐groups correlations showed that PA levels in pre‐pandemic and restrictions‐easing conditions were significantly correlated, both for youths (*r* = 0.70; *P* < 0.001) and parents (*r* = 0.65; *P* < 0.001) (Fig. 1).

**Figure 2:**
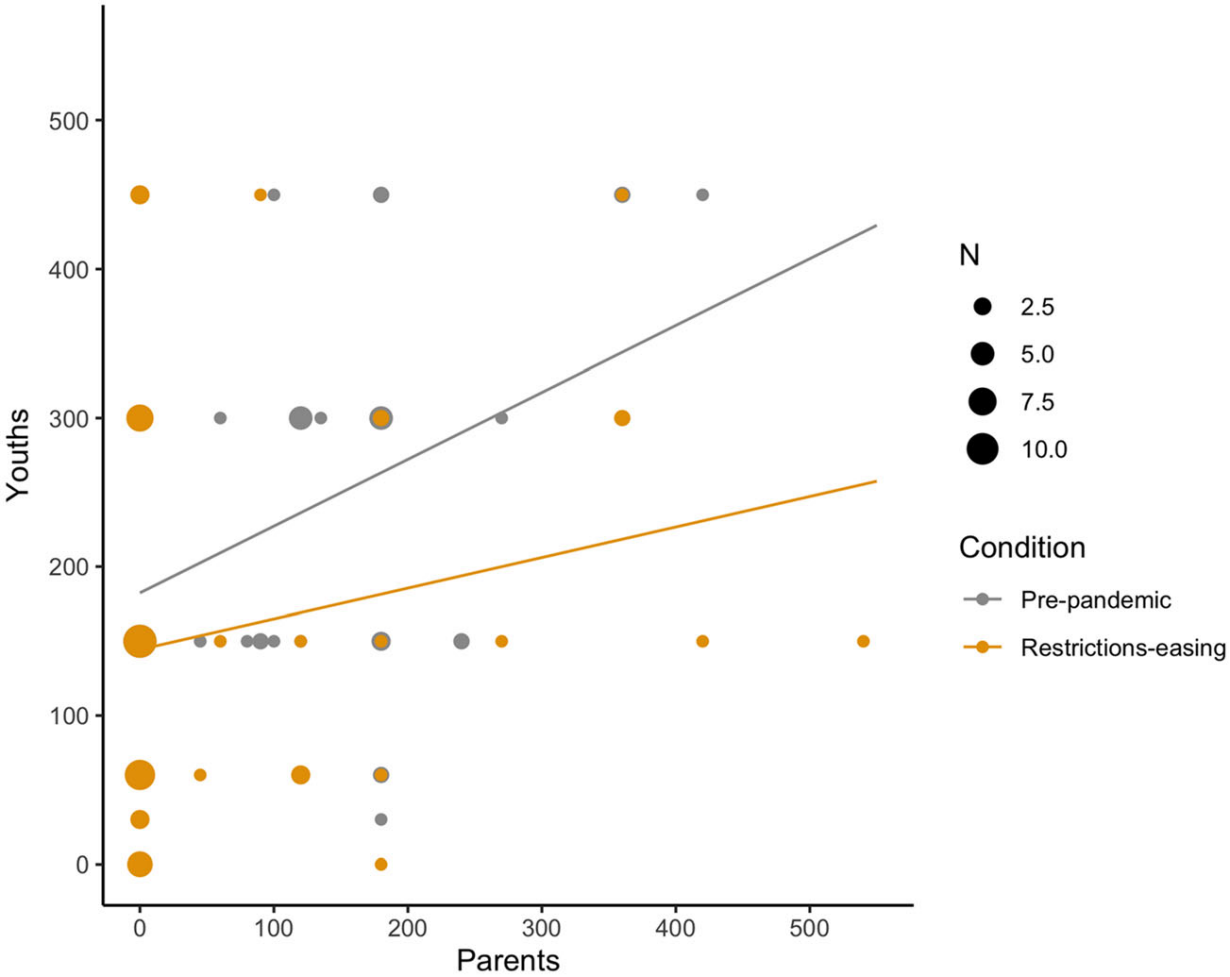
Correlations between PA levels (min/week) of youths and parents, in the pre‐pandemic (in grey) and restrictions‐easing (in orange) conditions. The size of the circles gets bigger for overlapped points. [Colour figure can be viewed at wileyonlinelibrary.com]

Furthermore, the between groups correlations highlighted a positive association between parents and youths' PA levels in the pre‐pandemic condition (*r* = 0.38; *P* = 0.004), but this relationship was no longer present in the restrictions‐easing phase (*r* = 0.19; *P* = 0.148) (Fig. 2).

## Discussion

The present study aimed to describe the changes in PA, SB and ST due to COVID‐19‐related restrictions in youths with DS, considering personal characteristics as independent variables, and to investigate the correlation with parental PA levels. The major findings indicated that PA levels decreased, and SB and ST increased due to the restrictions imposed by the government for preventing COVID‐19 diffusion, independently from gender, ID level and residential location. When the restrictions began to ease, SB and ST showed a return to the pre‐pandemic situation, a sign that at least a gradual resuming of daily routines happened and is likely still happening. In contrast, PA levels did not return to the habitual pre‐pandemic levels despite restrictions‐easing, neither for youths nor parents (Fig. 1); moreover, the positive association between youth and parental PA was also lost in the restrictions‐easing phase. Generally, the findings are in accordance with what was reported in a recent systematic review (Stockwell *et al*. 2021), underlying the need for public health strategies for promoting PA and decreasing SB during periods of reduced possibilities of movement (such as the pandemic), especially in populations with medical conditions that could be improved by PA practice, such as people with DS.

Average values for weekly PA of the participants with DS were 226.8, 105.2 and 158.4 min in the three different time‐points. In previous studies investigating levels of PA, measured for seven consecutive days using a triaxial accelerometer, it was found that the average PA undertaken by children and adolescents with DS was 153.1 (Whitt‐Glover *et al*. 2006) and 104.5 min/day (Shields *et al*. 2009), meaning more than 700 min/week. Our participants showed drastically lower rates of PA levels, partially explained by the different methods of measure (subjective proxy report vs. objective measures) and by the more accountable different age range of participants. Although the existing objective and subjective data on PA longitudinal development in youth with DS do not allow for a straightforward interpretation, it is likely that PA declines with age and that a large proportion of them do not meet the recommended amount of daily PA (Pitetti *et al*. 2013; Fox *et al*. 2019), similarly to the general population.

When looking at SB, in a recent review investigating its epidemiology in adults with ID (Melville *et al*. 2017), it was reported that sedentary time, objectively assessed, ranged from 522 to 643 min/day. Considering the underestimation that generally affects the self‐report techniques for SBs when compared with device‐derived measures (Prince *et al*. 2020), the participants of the present study showed daily average values that could be considered in line with those found for the ID population (mean values in the three time‐points were 382, 512 and 413 min/day, respectively). As a specific SB, the daily amount of ST deserves attention. In the three time‐points, participants of the present study reported an average screen‐based media volume of 186.8, 251.6 and 203.2 min/day, showing rates rather above the international recommendations for limiting screen exposure to <2 h/day (American Academy of Pediatrics: Committee on Public Education 2001; Tremblay *et al*. 2011). However, ST rates seemed in agreement with previous data concerning people with ID, reporting a prevalence higher than 50% of participants spending four or more hours per day watching TV (Melville *et al*. 2018).

While applying the WHO threshold for recommended PA in people with disability (Bull *et al*. 2020) to the present study's data, only 35.1% of participants respected the guidelines before the pandemic. This confirmed what was found by Shields *et al*. (2009), objectively reporting that only 42.1% of their participants with DS completed at least 60 min of moderate‐to‐vigorous PA each day. Moreover, in an Australian study, parents reported that less than one‐third of 208 youth with DS were active as defined by PA guidelines, although most of them participated in sports (Oates *et al*. 2011). Regarding this aspect, sports participation does not necessarily allow reaching sufficient levels of PA; besides, high levels of PA only partially abrogate consequences of SB (Ekelund *et al*. 2016). Furthermore, PA and ST are largely uncorrelated (Pearson *et al*. 2014), meaning that low levels of PA do not necessarily imply exceeding ST recommendation, and vice versa. Thus, it is important to consider PA and SBs in their complex interaction to explore the global health and quality of life of people with DS during the pandemic restrictions and beyond. Regrettably, a very low proportion of participants in this study reported concurrent achievement of PA and ST recommendations, with very low rates in the pre‐pandemic, during restrictions and in the restriction‐easing phase, respectively, of 21.1%, 5.3% and 10.5%. Accordingly, a recent investigation on the prevalence of Colombian adolescents meeting PA and ST guidelines showed similar rates (7.8%), hampered by the presence of sensory problems (Lopez‐Gil *et al*. 2021). The disability itself is a key barrier to PA participation and ST recommended dose. The overall trend registered for PA, SB and ST during the pandemic course corroborates the need for worldwide action plans regarding PA participation of people with disabilities (Martin Ginis *et al*. 2021), and further research is needed to investigate to what extent the magnitude of changes in PA and SB could have impacted the health and all the life domains of people with DS.

When detecting parent–child PA in order to better explain PA changes through the pandemic era, parents and their child's PA levels significantly correlated before the pandemic, confirming the literature for youths with developmental disabilities (Siebert *et al*. 2017); interestingly, this association is lost in the restrictions‐easing phase. It might be that parents during the restrictions have changed so many routines – experiencing greater anxiety, defeat, additionally family demands and new stressors (Doody & Keenan 2021) – that during the restrictions‐easing phase, they did not have the necessary time and energy for resuming personal PA (as represented in Fig. 1b). This corroborates the need for comprehensive PA promotion strategies directed to the whole family, not only to the person with a disability.

This study has several strengths. First, it provides, for the first time, an overview of the changes, from before the pandemic to the restrictions‐easing phase, passing through the full confinement period, of PA, SB and ST in youths with DS, analysing the prevalence of independent and concurrent meeting of PA and ST recommendations. Moreover, it provides an insightful element for parental influences. However, this study also presents some limitations. First, possible sources of error inherent in proxy‐report measures of PA, SB and ST compared with self‐report and objective measures should be considered; indeed, because of the pandemic‐related safety concerns, it was preferred to avoid personal contact and shared devices (such as accelerometers to collect objective PA data) with participants. Second, the fact that the variables were retrospectively assessed before the pandemic and during the restrictions could have enhanced the risk of recall and comparison bias. Further research is needed about people with DS and their caregivers to better understand the underlying mechanisms that explain the observed changes in PA and SBs. In particular, future research could aim to investigate the effects driven by interventions targeting factors at all social‐ecological model levels, all PA types (leisure, transport, household, education and occupational) and intensities, on this population's behaviours.

## Conclusions

The findings showed the negative impact of COVID‐19‐related restrictions on the lifestyle of youths with DS, with decreased PA and increased SBs and screen time. Particularly, PA did not recover in the restrictions‐easing phase, and the positive correlation with parental PA was lost. It is essential that we learn from the COVID‐19 pandemic how to preserve people with DS on account of their inherent vulnerability to the consequences of the restrictions used to manage the outbreak. The results support the importance of advancing the knowledge and sharing best practices within the DS population and their families/caregivers about PA and sports promotion to empower them to face such occurrences in the future.

## Source of funding

This research did not receive any specific grant from funding agencies in the public, commercial or not‐for‐profit sectors.

## Conflict of interest

The authors have no conflict of interest to declare.

## Data Availability

The data that support the findings of this study are fully available from the corresponding author upon reasonable request.
